# Soluble Expression of Disulfide Bond Containing Proteins FGF15 and FGF19 in the Cytoplasm of *Escherichia coli*

**DOI:** 10.1371/journal.pone.0085890

**Published:** 2014-01-20

**Authors:** Bo Kong, Grace L. Guo

**Affiliations:** Department of Pharmacology and Toxicology, Ernest Mario School of Pharmacy, Rutgers, The State University of New Jersey, Piscataway, New Jersey, United States of America; New England BioLabs, United States of America

## Abstract

Fibroblast growth factor 19 (FGF19) is the human ortholog of mouse FGF15, and both proteins function as an endocrine signal to regulate various liver functions. FGF15/FGF19 protein contains two disulfide bonds. It is unfavorable to form disulfide bonds in *Escherichia coli* (*E. coli*) cytoplasm because of the bacterial cytoplasmic reducing environment. Modification of the cytoplasmic reducing environment and/or co-expression of protein chaperones are common strategies to express disulfide bond containing proteins in *E. coli*. In the current study, we report a method to produce soluble FGF15/FGF19 protein in cytoplasm of *E. coli*. Several commercial available strains with the disruption of thiol-redox pathways, and/or co-expression of redoxase or refolding chaperones were used to develop this novel method for expression of FGF15/FGF19 in *E. coli*. Mutation of the thiol-disulfide bond reducing pathway in *E. coli* or N-terminal fusion of thioredox (TRX) alone is not enough to support disulfide bond formation in FGF15/19 proteins. However, TRX fusion protein improved FGF19 solubility in strains of thiol-redox system mutants. In addition, DsbC co-expressed in thiol-redox system mutants alone improved and further enhanced FGF19 solubility with combination of TRX fusion tag. The soluble FGF19 proteins were easily purified through Ni-NTA affinity chromatography and anion exchange chromatography, and the purified protein maintained its biological activities, confirmed by suppressing hepatic Cyp7a1 gene transcription in mice and by activating ERK1/2 signaling pathway in HepG2 cells. In contrast, soluble FGF15 protein in cytoplasm remained very low using these strategies. In summary, we have successfully developed a method to express functional FGF19 protein in prokaryotic cells, and this strategy may be adapted for the expression of other disulfide-containing proteins.

## Introduction

Fibroblast growth factor 19 (FGF19) is expressed in human liver and intestine and shows different tissue distribution from its mouse ortholog, FGF15, which is only expressed in the intestine [1]. However, both proteins function as enterohepatic hormones and they are secreted from the small intestine to regulate bile acid homeostasis in the liver. After being secreted into the portal circulation, FGF15/19 binds to its receptor, FGFR4, in the liver [2], [3] and activates downstream signaling pathways to suppress the transcription of the gene encoding cholesterol 7α-hydroxylase (Cyp7a1), the rate-limiting enzyme for bile acid synthesis [3]–[5]. Moreover, FGF15/19 has been shown to promote liver tumorigenesis [6], energy metabolism [7], insulin sensitivity [8], and liver regeneration [9], but the underlying mechanisms have not been fully clarified. Pure and functional proteins produced by an efficient method can provide a valuable tool to greatly improve the research of FGF19/15.

Due to easy handling, inexpensive cultivation and large-scale production, the *E. coli* bacterial system is a popular and well characterized prokaryotic host system for heterologous protein expression [10], [11]. However, the *E. coli* system also contains a few limitations, and expression of eukaryotic proteins in a bacterial system has been always challenging, especially when these proteins contain disulfide bonds [12], [13]. Disulfide bonds are very common in mammalian proteins and are crucial for proper protein folding, stability, and activity. They are formed into the covalent bond by the oxidation of thiol groups between two cysteine residues in the protein. Cytoplasm of *E. coli* is constantly maintained as a reducing environment, therefore in general, the *E. coli* cytoplasm is not favorable for the expression of proteins containing disulfide bonds, and the formation of disulfide-bond containing proteins in bacterial cytosol is unstable and normally forms inactive inclusion bodies. Therefore, additional *in-vitro* refolding is required to obtain biologically functional proteins. However, it is well known that *in-vitro* refolding of protein in inclusion bodies is often unpredictable and challenging [13]–[17], in addition to being time consuming and requiring a large amount of reagents. Overall, generation of protein in soluble form is the preferred choice.

Extensive efforts have been made to overcome these obstacles to improve soluble expression of different disulfide-bonded proteins in the cytosol of *E. coli*. Cysteines in the *E. coli* cytoplasm are actively kept reduced by pathways involving thioredoxin reductase and glutaredoxin [18]–[20], so one strategy is to alter these reducing pathways to change the cytoplasmic thiol-redox equilibrium environment. There are various types of commercially available mutant strains (AD494, Origami (Novagen), SHuffle (New England Biolabs) [18], [19], [21], which lack thioredoxin reductase (Δ*trx*), glutathione reductase (Δ*gor*) and/or glutathione biosynthesis (Δ*gshA*) and have been used successfully to improve the soluble expression of disulfide bond containing proteins that are unable to be expressed in the parent strain BL21(DE3) [13], [18]–[20]. Another widely adapted strategy is to co-express chaperones or oxidase to promote disulfide bond formation to stabilize the recombinant proteins in cytosol and improve protein expression. Thioredoxin (TRX, encoded by gene trxA) has been shown to enhance folding and disulfide bond formation when fused to the target protein in a thioredoxin reductase (ΔtrxB) mutant strain [13].

In the current work, a TRX fusion tag was attached to the N-terminus of the FGF19 and FGF15 protein, then the fusion proteins were expressed in various *E. coli* mutant strains, and soluble protein expression in bacteria cytoplasm were determined.

## Materials and Methods

### Ethics Statement

Mice were bred and maintained in the facility of the Laboratory of Animal Research at the University of Kansas Medical Center, and were housed in rooms under a standard 12-hr light/dark cycle with access to chow and water ad libitum. All protocols and procedures were approved by the Animal Care and Facilities Committee (ACFC) at the Rutgers University, The State University of New Jersey and are in accordance with the NIH and AALAC Guidelines (protocol #12-028). All experiments were performed with age-matched 10–16 weeks old male mice.

### Reagents

All chemicals were obtained from Sigma-Aldrich (Saint Louis, MO); HisPur Ni-NTA Chromatography Cartridge (5 mL) was from Pierce, Inc.; HisTrap HP, 1 ml cartridge and HiTrap Q HP anion exchange 5 ml cartridge were obtained from GE Life Sciences. The protein purification processes were performed on AKTA FPLC chromatography system with an automated fraction collector (GE Healthcare).

### Plasmid Construction

**Figure 1:**
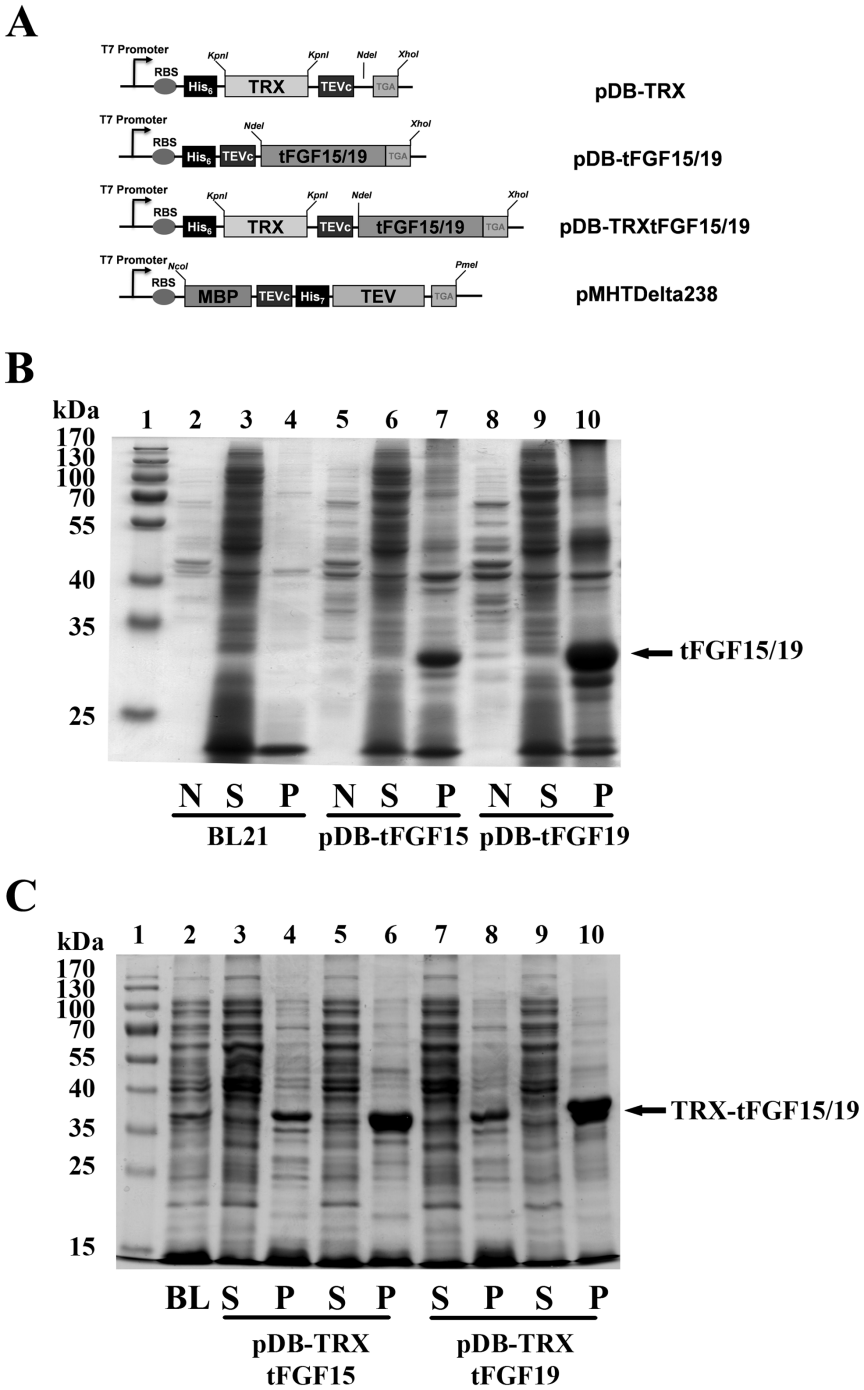
Construction and expression analyses of FGF15 and FGF19 Plasmids. A): Schematic maps of plasmids. Truncated FGF15 or FGF19 gene (without signal peptide) was located downstream from the T7 promoter, coding in frame with the TRX tag. *Nde*I and *Xho*I restriction enzyme sites are used to subclone the PCR fragments. His6 tag for purification and TEV protease site for fusion tag cleavage are indicated in the figure. B): SDS-PAGE analysis of FGF15 and FGF19 in *E. coli* strain BL21(DE3); non-induced total cell lysates (N), and the soluble (S) and insoluble fractions (P) were prepared as described in Methods. The molecular weight markers from Pierce (Rockford, IL) are also shown in gels. C): SDS-PAGE analysis of TRX-FGF15/19 in BL21(DE3) strain; the soluble (S) and insoluble fractions (P) before (Lane 3, 4, 7, and 8) and after IPTG induction (lane 5, 6, 9 and 10) were determined. Total cell lysates of BL21(DE3) strain (BL) were loaded as the control.

The FGF19 expression, Tobacco Etch Virus (TEV) protease (pMHTDelta238) [22], and pDB-TRX, plasmids were obtained from DNASU Plasmid Repository (dnasu.asu.edu/DNASU/Home.jsp). The FGF15 expression plasmid was previously described [23]. CDS fragments of FGF15 (26aa–219aa) or FGF19 (22aa–217aa) without the predicted signal peptide were PCR amplified using the following primers: FGF15-NdeI_F: 5′-ATGTCATATGCGTCCCCTGGCTCAGCAATC-3′, FGF15-XhoI_R: 5′-CCTGCTCGAGTCATTTCTGGAAGCTGGGACTC-3′; FGF19_NdeI_F: 5′-CGTGCATATGCGCCCCCTCGCCTTCTCGGA-3′; FGF19_XhoI_R: 5′-CCTGCTCGAGTTACTTCTCAAAGCTGGGAC-3′ (incorporated restriction enzyme sites underlined). The PCR amplicons were inserted into the pDB-TRX plasmid after the TRX sequence to generate plasmids, pDB-TRXtFGF15 and pDB-TRXtFGF19. The TRX sequence from pDB-TRX plasmids was deleted before cloning, followed by inserting the FGF15 or FGF19 PCR fragments into the plasmid through NdeI and XhoI sites to generate plasmids, pDB-tFGF15 and pDB-tFGF19, respectively. The schematic maps of plasmids are shown in Fig 1A. All plasmid DNA were verified by sequencing before transfection and protein expression.

### Protein Expression and Purification

*E. coli* strains of BL21 (DE3), Rosetta-gami 2 (EMD Biosciences, Madison, WI) and SHuffle T7 Express (New England Biolabs, Ipswich, MA) were used as expression hosts. The Rosetta-gami 2 strain contains chloramphenicol resistant plasmids of pRARE, which constitutively supplies tRNAs for seven rare codons. Plasmids of pRARE were isolated from Rosetta-gami 2 strain and co-transferred with FGF15/FGF19 expression plasmids to the SHuffle T7 Express strain.

Protein expression was performed as described previously [23]. Briefly, Kanamycin (100 µg/mL) was added to all media and chloramphenicol (34 µg/mL) was added to cultures of strains carrying pRARE plasmid. A single colony containing the FGF15 or FGF19 plasmids was inoculated in 10 ml LB media and cultured at 32°C overnight. 10 mL of overnight culture were used to inoculate 1L of LB medium supplemented with kanamycin and/or chloramphenicol next morning. Cells were cultured at 32°C to OD600 0.6, followed by induction by adding 0.4 mM isopropyl-β-D-thiogalactopyranoside (IPTG) at 20°C for 16 hrs before cell harvest. The cell pellets were harvested by centrifugation at 8000 g for 10 min, which were re-suspended in 50 ml cold PBS buffer (50 mM sodium phosphate, 150 mM NaCl, pH 7.5) with 20 mM imidazole. Cells were disrupted by intermittent sonication for 5 mins on ice using 30 s pulse and 30 s break for cooling, followed by centrifugation at 4°C for 20 mins at 15,000 g. The soluble fraction and insoluble pellet were used for SDS-PAGE analysis.

The retained soluble fractions were further processed for protein purification. Two steps of immobilized-metal affinity chromatography (IMAC) followed by ion exchange chromatography (IEX) were performed to purify the soluble proteins. Briefly, the supernatant was filtered (0.2 µm membrane) before applying to the Ni-NTA column pre-equilibrated with binding buffer (50 mM sodium phosphate, 20 mM imidazole, 150 mM NaCl, pH 7.5) via a 50-ml super-loop (Pharmacia). Elution by a 0–0.5M imidazole gradient in PBS buffer (pH 7.5) was performed. Fractions containing FGF15/FGF19 were pooled and exchanged by dialysis into PBS buffer (50 mM sodium phosphate, pH 7.5) at 4°C before cation exchange chromatography. The sample was concentrated after dialysis using an Amicon Ultra-15 filter device (MW cutoff at 10 kDa, Millipore) and then loaded onto a 5-ml Hi-Trap Q HP anion-exchange column equilibrated with PBS buffer followed by elution using a 0.1–0.5M NaCl gradient in PBS buffer (pH 7.5). Purity of the samples was assessed by SDS-PAGE. Total protein concentrations were determined with the Bradford assay by using bovine serum albumin as protein standard.

### Cleavage of TRX Containing Protein

Expression and purification of TEV protease were performed according to previously described methods [22]. The purified TEV protease was concentrated to 1 mg/ml and stored at −80°C in buffer (50 mM sodium phosphate, pH 7.5, 5 mM β-mercaptoethanol, 50% glycerol).

The purified proteins were incubated with TEV protease at a 1∶100 (w/w) ratio for 2 hrs at room temperature or overnight at 4°C to remove the N-terminal TRX tag linked to the tFGF19 (His6-TRXtFGF19) protein. 3 mM glutathione and 0.3 mM oxidized glutathione were added to the reaction buffer to preserve TEV protease activity. After digestion, the proteins were spun down at 15000 g for 10 min to remove protein aggregates. The supernatant was then loaded onto a HiTrap Chelating HP column equilibrated with PBS buffer to remove the His6-TRX tag, and recombinant protein tFGF19 was recovered in the flow-through fractions.

### FGF19 Protein Activity Assay

Biological activity of FGF19 was determined in both in-vivo and in-vitro models. Doses of 10 µg and 100 µg protein/kg mouse body weight were used to treat C57BL6/J mice through tail vein injection. Two hrs later, Livers were collected and Cyp7a1 gene expression was determined by real time-qPCR [3], [23].

HepG2 cells were cultured in DMEM media with 10% FBS to 80% confluence, then treated with 10 ug per ml FGF19 protein for indicated periods or for 30 min with various protein concentrations. Cells were lysed with protease and phosphatase inhibitors in RIPA buffer (50 mM Tris-HCl pH 7.4, 150 mM NaCl, 1% NP-40, 0.5% sodium deoxycholate, 0.1% SDS). Western blot was performed on these samples using the antibodies against phosphor−/total- ERK (Cell Signaling Technology) [3]. The β-actin protein was used as the loading control.

## Results

### TRX Fusion Tag did not Improve FGF15/19 Solubility in BL21 (DE3) Strain

The cDNAs encoding truncated FGF15 (26–219) or FGF19 (22–217) protein were cloned into pDBHis plasmids without N-terminus TRX fusion (Fig. 1A) and transferred into the BL21(DE3) strain. BL21(DE3) maintains a normal reducing environment in the cytosol so that disulfide formation is not supported it. Previous studies of FGF15 and FGF19 protein have shown that the two disulfide bonds are essential to maintain protein structure and activity [23]–[25]. Therefore, after IPTG induction, we evaluated the expression level and solubility of each construct on SDS-PAGE (Fig. 1B and 1C). All FGF15/19 and TRXtFGF15/19 proteins were expressed as their predicted molecular size, but as expected, they were only expressed in the insoluble fraction as inclusion bodies (Fig. 1B and 1C). The TRX fusion tag wasn’t able to improve FGF15 and FGF19 protein soluble expression in the BL21(DE3) *E. coli* strain.

### TRX Fusion Tag Improved Expression of Soluble FGF19 in Rosetta-gami Strain

**Figure 2:**
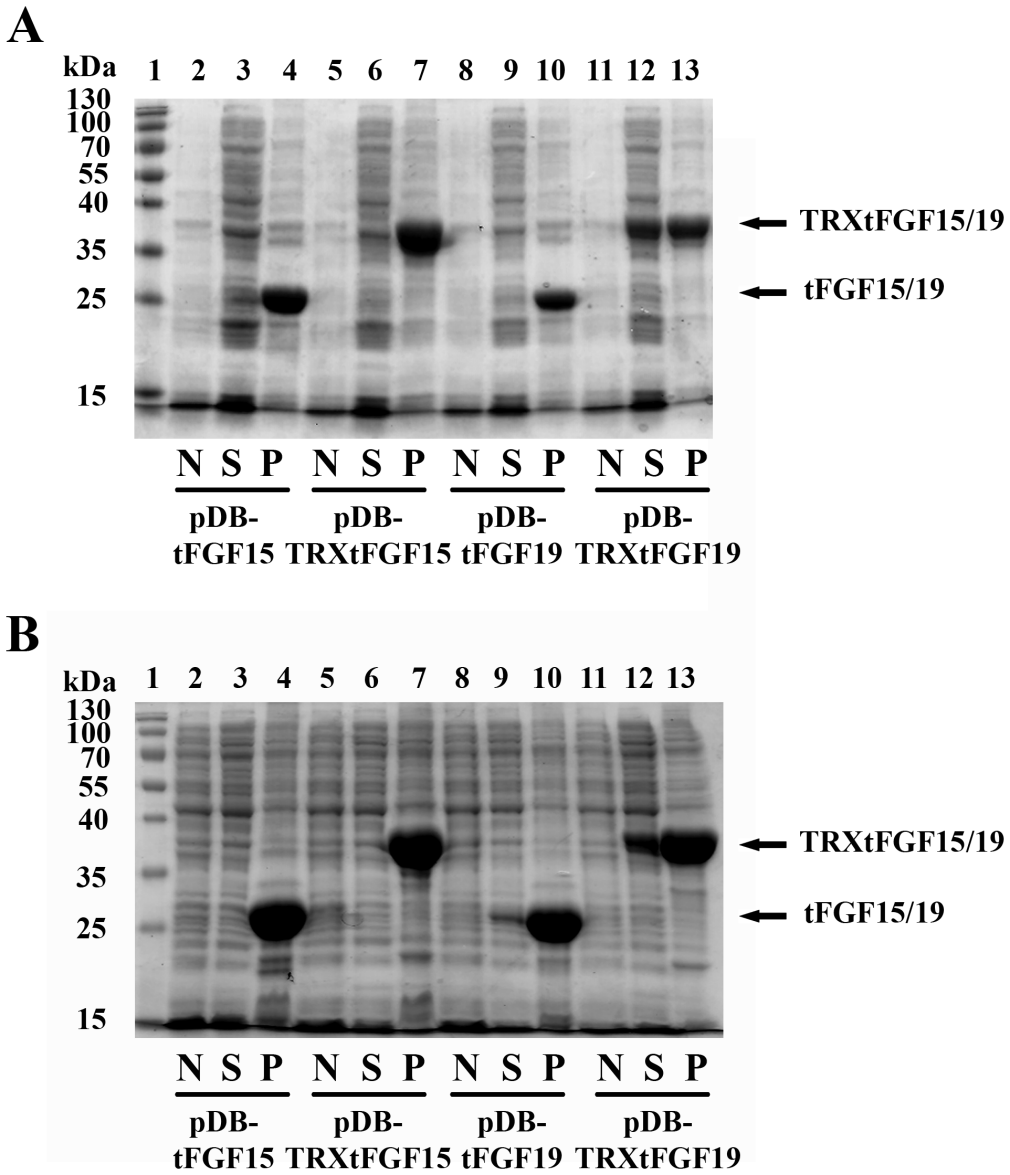
SDS-PAGE analysis of FGF15/19 and TRXtFGF15/19 expression in *E. coli* strains of Rosetta-gami 2 (A) and SHuffle T7 (B), showing the effect of TRX fusion protein, co-expression of Dsb and disruption of cytosolic reducing environment on the expression of soluble FGF15/19 in the cytoplasm. Total cell lysates without IPTG induction (N), the soluble (S) and insoluble fractions (P) from IPTG induced cell lysates prepared as described in Methods. The arrows indicate the predicted positions according to the protein size. Lane 1: protein markers; Lane 2: FGF15 without induction; lane 3: soluble FGF15 fraction after induction; lane 4: insoluble FGF15 fraction after induction; Lane 5: TRXtFGF15 without induction; lane 6: soluble TRXtFGF15 fraction after induction; lane 7: insoluble TRXtFGF15 fraction after induction; Lane 8: FGF19 without induction; lane 9: soluble FGF19 fraction after induction; lane 10: insoluble FGF19 fraction after induction; Lane 11: TRXtFGF19 without induction; lane 12: soluble TRXtFGF19 fraction after induction; lane 13: insoluble TRXtFGF19 fraction after induction.

Rosetta-gami 2 is a commercially available strain from Novagen that carries mutations for the reductases (*trxB* and *gor*) for disulfide bond [13], [18]. In the absence of TRX fusion tag, both FGF15 and FGF19 proteins were over-expressed after IPTG induction, but only expressed as insoluble inclusion bodies, and no soluble FGF15 and FGF19 protein could be found expressed in cytosol after overnight induction (Fig. 2A, lane 3 and 9). When combined with a TRX fusion partner, soluble TRX-FGF19 protein could be detected in the cytosol fraction after IPTG induction (Fig. 2, lane 12); however, there was no soluble TRX-FGF15 (Fig. 2A, lane 6). The TRX tag enhanced the solubility of FGF19 protein in the cytoplasm of Rosetta-gami 2 strain, but did not improve the solubility of FGF15 protein.

### Co-expression of Dsb and TRX further Enhanced Cytosol FGF19 Solubility in SHuffle T7 Express Strain

The SHuffle T7 Express strain is from New England Biolabs, which carries cytoplasmic expression of chaperone DsbC in addition to trxB/gor mutations [21]. After IPTG induction, most of the FGF15 and FGF19 protein was induced as inclusion bodies and only existed in the insoluble fraction, however there was some FGF19 protein expressed in the soluble fraction (Fig 2B, lane 9), but no obvious soluble FGF15 expressed in the cytoplasm (Fig 2B, lane 3).

In addition, we also examined protein expression and solubility of FGF15 and FGF19 in the presence and absence of TRX fusion tag after IPTG induction. Soluble TRXtFGF19 proteins were greatly increased compared to FGF19 protein (Fig 2B, lane 12), and the relative amount of soluble protein increased when compared to the TRXtFGF19 protein expression in Rosetta-gami strain. However, there was still no soluble TRXtFGF15 protein expressed (Fig 2B, lane 6).

### Purification of Recombinant FGF19 Protein

**Figure 3:**
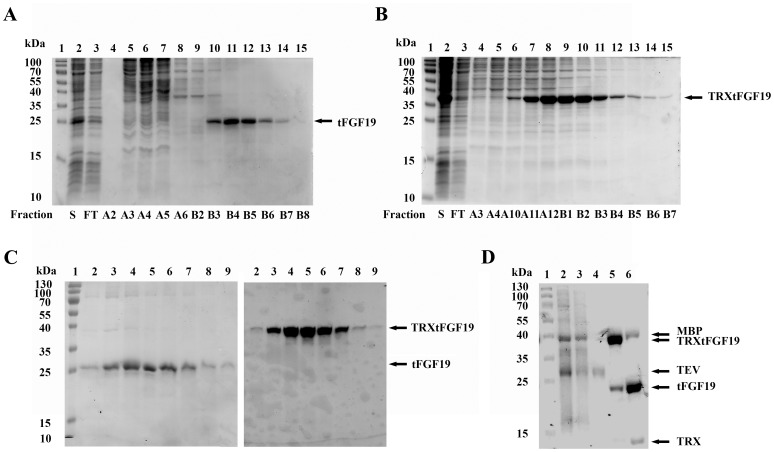
Purification of soluble FGF19 and TRXtFGF19 proteins from *E. coli* cytosol lysates. Purification of soluble FGF19 (A) and TRXtFGF19 (B) from cell lysates by Ni-NTA affinity chromatography with AKTA FPLC system. Lane 1: protein markers; Lane 2: lysate (soluble fraction); Lane 3: flow through; Lane 4 to 15: elution fractions. (C) The second cycle of cation exchange chromatography (HiTrap Q column) of elution peak pool from IMAC column. Lane 1: Molecular weight. Lane 2 to 9: fractions contain purified FGF19 (left panel) and TRXtFGF19 (right panel). (D) Purification of TEV protease produced from BL21(DE3) strain by IMAC chromatography with AKTA FPLC system. Lane 1: protein markers; Lane 2: Soluble fraction of total cell lysate; Lane 3: Ni-NTA column flow-through; Lane 4: purified TEV protease; Lane 5: purified TRXtFGF19; Lane 6: TRXtFGF19 protein after TEV protease digestion. Arrow indicates the protein size according to their predicted molecular weight.

The AKTA FPLC purification system was used to purify the soluble FGF19 and TRX-FGF19 protein from the SHuffle T7 cell lysate. Two-step purification consisting of IMAC followed by anion exchange chromatography was performed to purify the soluble proteins. Figs A, B and C are the representative SDS-PAGE gel analysis for the FPLC fraction. After IMAC purification (Fig. 3A, 3B), the contaminants couldn’t be resolved, especially in the TRXtFGF19 protein (Fig. 3B). However, after the second step of anion exchange chromatography, more than 90% purity of FGF19 (Fig. 3C left) and TRX-tFGF19 (Fig. 3C right) was reached, and only one band was detected on SDS-PAGE gels stained with Coomassie blue R250 (Fig. 3C). The yields of purified protein in the T7 SHuffle strain were about 0.5 mg for FGF19 and 2 mg for TRXtFGF19 per liter of culture media. For the FGF15 protein, only a small amount of TRXtFGF15 could be detected in SHuffle T7 strains using IMAC column enrichment, and the final yield was very low because of the protein loss during the purification process.

One step of IMAC chromatography to purify the TEV protease harvested 90% pure protein (Fig. 3D, lane 4), Moreover, the purified TEV protease maintained its activity, as shown by its ability to cleave the protease site between the TRX fusion tag and the C-terminus tFGF19 moiety in TRXtFGF19 protein (Fig. 3D lane 6).

### Cleavage of TRXtFGF19

**Figure 4:**
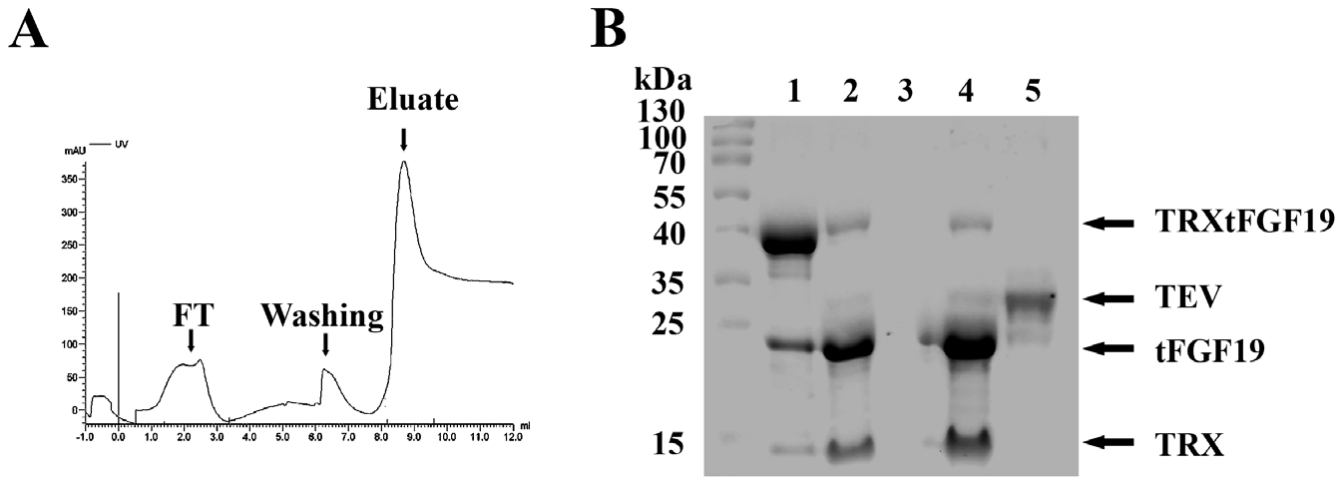
Purification of FGF19 protein from TRXtFGF19 after TEV protease digestion. (A) Elution profile of Ni-NTA IMAC chromatography. FGF19 Protein didn’t exist in flow-through (FT) fraction as expected, but bound to the column and competitively eluted by increased imidazole concentration. (B) SDS-PAGE analysis the TRXtFGF19 cleavage and tFGF19 purification by Ni-NTA column. Lane 1: purified TRXtFGF19 protein; Lane 2: TRXtFGF19 digested by TEV protease; Lane 3: Flow-through from Ni-NTA column; Lane 4: eluate from Ni-NTA column using 200 mM imidazole; Lane 5: purified TEV protease as control.

After purification, the TRX tag was removed from the recombinant Fgf19 fusion proteins by Tobacco Etch Virus protease (TEV) cleavage. Fig.4 shows a typical result for an assay of TEV protease with the protein substrate TRXtFGF19. Some proteolysis was observed from the purified TRXtFGF19 protein even without TEV protease digestion (Fig.4 lane 1). After TEV protease digestion, most of the TRX-tFGF19 protein was cleaved (Fig.4 lane 2).

The N-terminal TRX fusion tag was released from the remainder of the fusion protein by proteolysis as shown by the presence of two additional protein bands, TRX (MW 13 kDa) and tFGF19 (MW 23 kDa). The protein mix after digestion was applied onto Ni-MTA column (Fig. 4A), and TRX fusion tags were supposed to bind the column while pure FGF19 protein could be collected from flow-through. Surprisingly, only minor FGF19 protein could be detected in the flow-through solution. All FGF19 protein were stacked and bound to the column (Fig. 4B, lane 3) and were eluted by imidazole solution with the other his-tagged protein, TRX, TEV and non-cleaved TRXtFGF19 (Fig. 4B, lane 4).

### Recombinant FGF19 Protein Maintained its Activity

**Figure 5:**
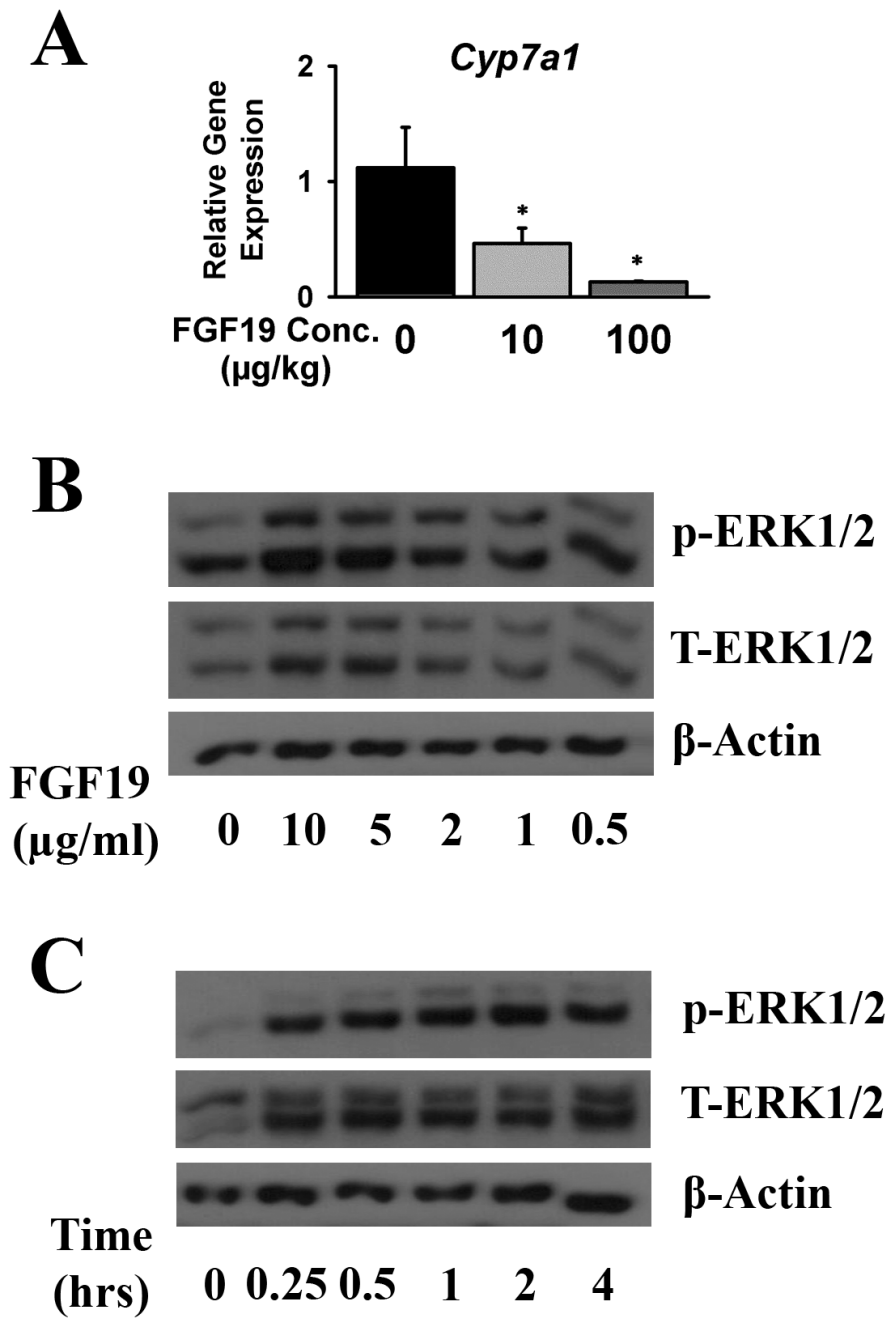
Biological activity of the purified FGF19 proteins. (A) Hepatic Cyp7a1 mRNA levels in mice 2 hrs after tail vein injection of vehicle (saline), FGF19 (10 µg/kg and 100 µg/kg body weight) *P<0.05, significant difference compared to vehicle-treated group. (B) Dose-dependency in activating ERK1/2 kinase in HepG2 cells by recombinant FGF19 for 2 hrs. (C) Time course in activating ERK1/2 kinase in HepG2 cells by 10 µg/ml FGF19.

Since tFGF19 could not be separated from the other protein after cleavage, recombinant FGF19 protein produced from a construct of pDB-tFGF19 in T7 SHuffle strain was used to test the protein activity *in vivo* and *in vitro*. FGF19 has been shown to bind to its receptor, FGFR4, in the liver and thereafter activate its downstream MAPK signaling pathways and eventually suppress Cyp7a1 gene expression [1], [3]. As low as 10 ug/kg dose of recombinant FGF19 significantly suppressed Cyp7a1 gene expression in mice (Fig. 5A), and the suppression effect was in a dose-dependent manner, which was similar to the refolded FGF15 protein previously reported [23].

The biological activity of the recombinant tFgf19 protein was also confirmed on HepG2 cells *in vitro* (Fig. 5B and 5C). Increasing amounts of FGF19 activated ERK1/2 pathways revealed by increased phosphor-ERK1/2 (Fig. 5B). In detail, as low as 1 µg/ml dosage of FGF19 activated ERK1/2 phosphorylation. Furthermore, the ERK1/2 pathway was activated as early as 15 mins after FGF19 treatment (Fig. 5C). All these data indicate that the soluble FGF19 protein expressed in *E. coli* cytosol was biologically active.

## Discussion

The *E. coli* prokaryotic system is still the most popular protein expression system because of the well-defined mechanisms and low cost. However, we face some challenges using the *E. coli* system to express mammalian proteins [10], [11], [26]. First, high-yield production of mammalian proteins in *E. coli* often leads to improper protein folding, which results in the insoluble proteins that form inactive aggregates known as inclusion bodies [26]. Second, *E. coli* lacks the protein post-translational mechanism, so it is only suitable for expression of protein without post-translational modifications (glycerilation, sumolyation, et al.) [10], [26]. Third, *E. coli* does not allow disulfide bond formation in the cytoplasm, and all its own proteins containing disulfide bonds are secreted into periplasm and folded there [11], [27].

Previous studies have shown no post-translational modification in mature FGF15 and FGF19 proteins [23], [24], indicating that the bacterial system could be used for expression of both proteins. However, both proteins contain two disulfide bonds, suggesting that they may be expressed as insoluble inclusion bodies due to the lack of ability to form disulfide bond in the reducing environment of *E. coli* cytoplasm. With the modification of the cytoplasmic reducing environment and co-expression of fusion protein TRX chaperones, soluble FGF19 protein can be expressed in bacterial cytosol, and this result is consistent with a previous finding showing that thioredoxin can improve disulfide bond formation in a reducing background [13]. Interestingly, co-expression of DsbC alone in the reducing environment of the cytoplasm was sufficient to improve FGF19 solubility. Disulfide isomerase (DsbC) is a periplasmic enzyme that catalyzes the isomerization of disulfide bonds. DsbC has been found to function as a chaperone to improve the solubility of disulfide bonds containing proteins in *E. coli* cytoplasm [28]–[30]. This could also explain why TRX fusion protein in the presence of DsbC would further improve FGF19 solubility. However, modification of the reducing environment and co-expression with these chaperones did not help produce satisfactory quantities of soluble FGF15 protein, indicating that other factors may be critical for its soluble expression. Moreover, it’s well known that every species has codon usage bias, and differences in codon usage can impede translation due to shortage or lacking for one or more tRNAs when heterologous proteins are overexpressed in *E. coli* [20]. Plasmids of pRARE2 from Rosetta-gami 2 cells, which supplies tRNAs for seven rare codons (AUA, AGG, AGA, CUA, CCC, GGA, and CGG), were isolated and co-transfected with the FGF15 and FGF19 expression plasmids into SHuffle T7 *E. coli* strains, but the results showed that overexpression of the rare tRNAs had no effect on the FGF15 and FGF19 solubility in the cytoplasm (data not shown), indicating that codon bias is not the major reason to prevent FG15 soluble expression. More studies need to be performed to determine the mechanism.

Because both FGF15 and FGF19 have been shown to suppress the expression of the Cyp7a1 gene that is critical in bile acid synthesis through activating its receptor, FGFR4, they were predicted to have the same features. However, the FGF15 protein only shares 50% sequence homology with FGF19; moreover, our results showed that it’s much easier to express soluble FGF19 protein or refold FGF19 protein from inclusion bodies than those for FGF15 protein, indicating that there might be a greater difference between these two proteins than expected. The crystal structure of FGF19 has been well characterized [24], [31],but the structure of FGF15 protein has not been determined, so it will benefit the researches in this area to figure out the limiting factors for FGF15 protein soluble expression in *E. coli*, and then adapt certain strategies to improve its solubility.

TRX fusion tag with the N-terminus 6XHis tag is located in the N-terminus and in frame with the coding sequences of gene FGF19 (His6-TRX-FGF19). The fusion protein (His6-TRX-FGF19) is purified by immobilized metal affinity chromatography (IMAC) on Ni-NTA resin and then cleaved *in vitro* with TEV protease (His6-TEV protease), which recognizes and cleaves the enzyme site between the TRX fusion tag and FGF19. We expected the His6 tagged TRX fusion part to bind to the Ni-NTA column and the released FGF19 protein to flow through. Surprisingly, most of the FGF19 protein bound to the column even without the His tag and didn’t appear in the flow-through fraction. Two possible reasons might explain this phenomenon. First, the FGF19 proteins were bound to the resin matrix nonspecifically; and second, the FGF19 proteins bound to the fusion protein TRX so that FGF19 could not easily disassociate with His6-TRX even with the cleavage at the TEV protease site during the purification process. However, increasing the NaCl salt concentration to up to 1 M could not prevent the FGF19 protein from binding to the TRX fusion protein and couldn’t reduce hydrophobic interactions and dissociate the FGF19 protein from the column after the digestion solution was loaded onto the Ni-NTA column (Fig. 4 lane 3). FGF19 eluted with the other His6 tagged protein with the increased imidazole concentration (Fig. 4 lane 4), indicating that the interaction between FGF19 and Ni-NTA was His6 tag related, rather than a nonspecific hydrophobic binding, so we believed that FGF19 bound Ni-NTA tightly through its interaction with the TRX fusion protein. More details need to be further investigated.

In conclusion, though we need to further work on soluble FGF15 protein production, we have developed a simple and effective method for producing quantities of soluble FGF19 protein in the *E. coli* system, and we believe that the strategy presented in this study could also be adapted to produce other valuable mammalian disulfide bond containing proteins in *E. coli* expression system.
